# Age Related Macular Degeneration and Total Hip Replacement Due to Osteoarthritis or Fracture: Melbourne Collaborative Cohort Study

**DOI:** 10.1371/journal.pone.0137322

**Published:** 2015-09-10

**Authors:** Elaine W. Chong, Yuanyuan Wang, Liubov D. Robman, Khin Zaw Aung, Galina A. Makeyeva, Graham G. Giles, Stephen Graves, Flavia M. Cicuttini, Robyn H. Guymer

**Affiliations:** 1 Centre for Eye Research Australia (CERA), The University of Melbourne, Royal Victorian Eye and Ear Hospital, Melbourne, Victoria, Australia; 2 Singapore Eye Research Institute, Singapore National Eye Centre, Singapore, Singapore; 3 Department of Epidemiology and Preventive Medicine, School of Public Health and Preventive Medicine, Monash University, Alfred Hospital, Melbourne, Victoria, Australia; 4 Centre for Epidemiology and Biostatistics, Melbourne School of Population and Global Health, The University of Melbourne, Melbourne, Victoria, Australia; 5 Cancer Epidemiology Centre, Cancer Council Victoria, Melbourne, Victoria, Australia; 6 Department of Orthopaedic, Repatriation General Hospital, Adelaide, South Australia, Australia; 7 Australian Orthopaedic Association National Joint Replacement Registry, Discipline of Public Health, School of Population Health & Clinical Practice, University of Adelaide, Adelaide, South Australia, Australia; Institut de la Vision, FRANCE

## Abstract

Osteoarthritis is the leading cause of total hip replacement, accounting for more than 80% of all total hip replacements. Emerging evidence suggests that osteoarthritis has a chronic inflammatory component to its pathogenesis similar to age-related macular degeneration. We evaluated the association between age-related macular degeneration and total hip replacement as proxy for severe osteoarthritis or fractured neck of femur in the Melbourne Collaborative Cohort Study. 20,744 participants had complete data on both age-related macular degeneration assessed from colour fundus photographs taken during 2003–2007 and total hip replacement. Total hip replacements due to hip osteoarthritis and fractured neck of femur during 2001–2011 were identified by linking the cohort records to the Australian Orthopedic Association National Joint Replacement Registry. Logistic regression was used to examine the association between age-related macular degeneration and risk of total hip replacement due to osteoarthritis and fracture separately, adjusted for confounders. There were 791 cases of total hip replacement for osteoarthritis and 102 cases of total hip replacement due to fractured neck of femur. After adjustment for age, sex, body mass index, smoking, and grouped country of birth, intermediate age-related macular degeneration was directly associated with total hip replacement for osteoarthritis (odds ratio 1.22, 95% CI 1.00–1.49). Late age-related macular degeneration was directly associated with total hip replacement due to fractured neck of femur (odds ratio 5.21, 95% CI2.25–12.02). The association between intermediate age-related macular degeneration and an increased 10-year incidence of total hip replacement due to osteoarthritis suggests the possibility of similar inflammatory processes underlying both chronic diseases. The association of late age-related macular degeneration with an increased 10-year incidence of total hip replacement due to fractured neck of femur may be due to an increased prevalence of fractures in those with poor central vision associated with the late complications of age-related macular degeneration.

## Introduction

Hip osteoarthritis (OA) and fractured neck of femur (#NOF) are both major public health problems[1,2]. Hip OA is one of the commonest and most disabling forms of OA with one in four individuals at risk of developing symptomatic hip OA in their lifetime[3]. Hip OA leads to loss of mobility and severe restriction in activity, significantly decreasing quality of life[4]. Currently, hip OA is the leading cause of total hip replacement (THR), accounting for 88% of all THR in Australia[5]. Although #NOF is a common clinical problem in the elderly, for whom it is a major cause of morbidity and mortality[1], it accounted for a small percentage (4%) of all THR in Australia[5]. It is estimated that falls in the elderly lead to injury 60% of the time, most are minor (50%), but 1 in ten results in more serious injuries (5%) and fractures (5%). Hospital admission for these injuries are often the precipitating event in 40% of admissions to long term institutional care[6] and only half of those with a hip fracture regain the same degree of mobility that they had before[7]. In Australia, hip fractures accounted for approximately 0.9% of government health services expenditure for 1995–1996[8]. The number of THR for the treatment for end-stage hip diseases has increased by 45% in the last decade, with 27,508 procedures performed in Australia, in 2012[5].

Age-related macular degeneration (AMD) is a progressive degenerative disease, which causes central vision loss, resulting in difficulty with reading, recognizing faces and driving. AMD is one of the leading causes of blindness and visual impairment in Australia and the developed world, and presents a major burden on healthcare resources[9]. Typically, visual acuity is often normal or mildly affected in early or intermediate AMD (where drusen accumulate in the macula, sometimes along with pigmentary changes). Late AMD typically results in significant central vision loss, due to either neovascular AMD (‘wet’ AMD) or geographic atrophy (‘dry’ AMD), but peripheral vision for navigation is usually preserved.

It is now well accepted that AMD is a chronic inflammatory disease with strong associations found with genes in inflammatory pathways[10–12]. OA is also regarded as a chronic low grade inflammatory disease[13] associated with increased C-reactive protein levels[14]. Evidence also suggests that genes associated with the inflammatory pathways are associated with OA[15]. As chronic systemic inflammation is thought to play an important role in the pathogenesis of both AMD and OA[11,12,16,17], we hypothesized that there might be an association between AMD and OA because of common inflammatory pathways. To date, we are unaware of studies evaluating the associations between AMD and OA. With THR being one of the most commonly performed joint replacement surgery in Australia, and hip OA accounting for the large majority of all THR surgeries (> 80%), we used THR data from the Australian Orthopedic Association National Joint Replacement Registry (AOA NJRR) which records joint replacements performed across the health system in Australia, as a surrogate measure for severe hip OA, to address this question.

Registry data on THR due to #NOF were also analyzed with respect to AMD. To date, two studies have directly evaluated AMD and its association with hip fractures. One study found an 11% increase in the four-year odds of hip fracture only with geographic atrophy but not neovascular AMD, while the other study found a 9% increase in hip fracture with newly diagnosed AMD (AMD type was not well defined)[18,19]. In the present study we also evaluated whether AMD was associated with #NOF, particularly as late AMD leads to vision loss and vision loss is known to increase the risk of falls and fractures[20].

## Patients and Methods

### Study participants

**Fig 1:**
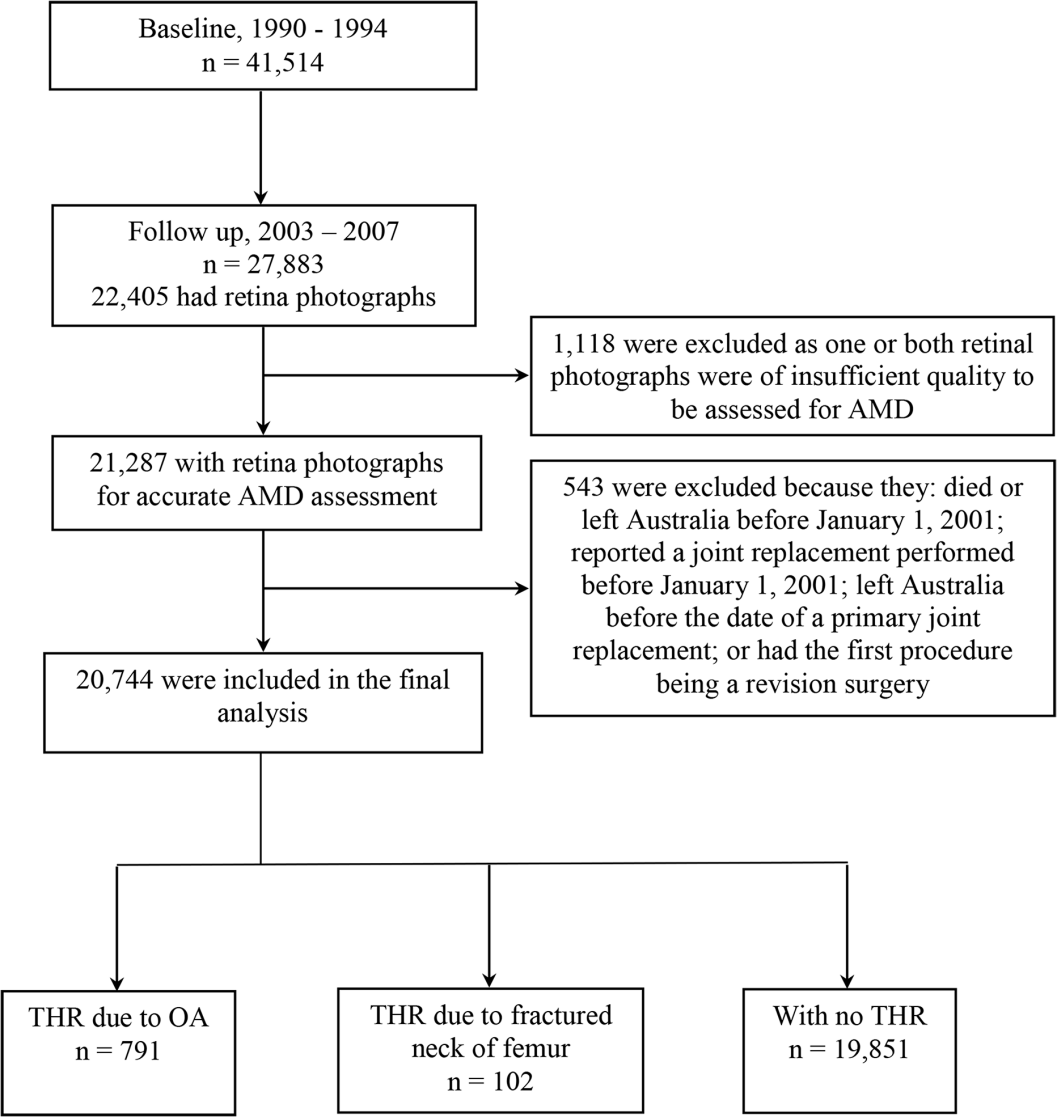
Study flowchart.

The Melbourne Collaborative Cohort Study (MCCS) recruited 41,514 participants (24,469 women) majority aged between 40–69 years at baseline (1990–1994) to examine links between lifestyle risk factors and chronic diseases[21]. Participants were recruited via Electoral Rolls (registration to vote is compulsory in Australia), advertisements, and community announcements in the local media. Of the total sample, 27,883 (67%) attended a follow-up examination between 2003 and 2007. Of these, 22,405 (80%) participated in colour fundus photography, with 21,287 with photographs of good quality to enable image-based classification of their AMD status. During the time period of this study there were no anti-vascular endothelial growth factor (VEGF) treatments available for wet AMD hence late neovascular AMD would have been readily seen on a colour photograph. Of the participants with graded fundus images, a further 543 participants were excluded because they had died or had left Australia before 1 January 2001, had joint replacement performed before 1 January 2001 (THR data collection only started after this date), or had the first recorded procedure being a revision surgery. The remaining cohort of 20,744 was included in the final analysis (Fig 1). The Cancer Council Victoria’s Human Research Ethics Committee approved the study. The Human Research Ethics Committee of the Royal Victorian Eye and Ear Hospital approved the AMD study protocol. The research adhered to the tenets of the Declaration of Helsinki. All participants gave written consent to participate and for the investigators to obtain access to their medical records.

### AMD assessment

At follow-up from 2003 to 2007, digital non-stereoscopic 45° macular photographs of both eyes were taken with a Canon CR6-45NM non-mydriatic retinal camera. Retinal photographs were graded for AMD by two experienced graders, using “OptoLite/OptoMize Pro” software (Digital HealthCare Image Management Systems, Cambridge, UK), and classified according to the Clinical Classification of AMD[22]. Quality control procedures for the photographs in the MCCS have been described elsewhere[23].

Participants were allocated to a single AMD category according to the more advanced visible changes on the macula images of the worse affected eye. Participants with images of insufficient quality in one or two eyes were excluded, as AMD status in the worse eye could not be reliably determined; this excluded persons with very small pupils or visually significant cataracts. Early AMD was defined as presence of one or more drusen 63 to 124μm in size (without pigmentary change); Intermediate AMD was defined as the presence of one or more drusen ≥125 μm in size (with or without pigmentary abnormalities), or one or more drusen 63 to 124μm with pigmentary abnormalities, in a 6000-μm diameter grading grid centered on the fovea, in the absence of late AMD in either eye (geographic atrophy or neovascular AMD)[22]. Geographic atrophy was defined as an area of retinal pigment epithelium (RPE) depigmentation larger than or equal to 175μm, which was roughly round or oval with sharp margins and visible underlying choroidal vessels. Neovascular AMD was defined as serous and/or haemorrhagic retinal pigment detachments, and /or subretinal neovascular membrane, and/or fibrous scarring in the absence of other retinal-vascular conditions. Participants with drusen <63μm and those without retina pathology that precluded fundus grading for AMD were used as controls.

### Identification of total hip replacement due to hip OA or fracture

To define a group of participants with severe hip OA or #NOF, we were able to access the AOA NJRR, which also records the reason for the hip replacement surgery. Australia has a publicly funded universal health insurance system (Medicare), therefore the entire Australian population has complete access to quality health care service including joint replacements. Therefore hip replacement as recorded in the AOA NJRR provides validated and complete data on the incidence of severe hip OA or #NOF, requiring hip replacement surgery. The implementation of the AOA NJRR commenced in 1999 and was completed nationally by mid 2002. Victorian data collection commenced on 1^st^ January 2001, therefore persons with fundus images but who had THR prior to this date were excluded from this study. The Registry had detailed information on the joint replacement prostheses, patient demographics, the indication for joint replacement, whether it was a primary joint replacement or a revision surgery and the type of revision[24]. Although contribution to data collection for the registry is voluntary, the registry receives cooperation from all hospitals and surgeons undertaking joint replacement surgery. In addition, the AOA NJRR validates its data against State and Territory Health Department unit record data using a sequential multilevel matching process. Following the validation process and retrieval of unreported records, the Registry collects an almost complete set of data relating to hip and knee replacement in Australia[24].

Identifying information for MCCS participants, including first name, last name, date of birth, and sex, were provided to the AOA NJRR in order to identify those MCCS participants who had had a joint replacement performed between 1 January 2001 and 31 December 2011. Data matching was performed on these data provided using the Freely Extensible Biomedical Record Linkage (Febrl) system. Exact matches were identified and probabilistic matches were reviewed. Using the Febrl system four matching runs were performed. The first run was an exact match on all the data fields comprising first name, last name, date of birth and gender. Subsequent runs were based on probabilistic or ‘fuzzy’ matching criteria using the remaining unmatched procedures. This allows for matches where the data fields may be close but not necessarily equal. Three such probabilistic matches were considered: (1) Procedures with the same date of birth. A match was determined on the quality of the remaining data fields; (2) Procedures with the same postcode and first name. A match was determined on the quality of the remaining data fields. (3) Procedures with the same first and last name. A match was determined on the quality of the remaining data fields. Of the procedures matched to the MCCS data, 91.0% were an exact match, i.e. first name, last name, date of birth and gender. The data linkage of the AOA NJRR with the MCCS was approved by The Cancer Council Victoria’s Human Research Ethics Committee and the Monash University Human Research Ethics Committee.

THR for OA or #NOF was defined as any THR in combination with a contemporaneous diagnosis of hip OA or #NOF between January 2001 and December 2011, as recorded in the AOA NJRR. Data on bone mineral density or cause of #NOF were not available in this database. The MCCS was not primarily designed to assess joint disease in its baseline questionnaires; however using the AOA NJRR data, the prevalence of severe OA or #NOF requiring joint replacement can be suitably captured. But OA affecting other joints that do not commonly undergo joint replacement surgery was not captured in this study.

### Assessment of demographic, lifestyle and anthropometric factors

At baseline, a structured interview schedule was used to obtain demographic and lifestyle information including date of birth, country of birth, smoking, alcohol consumption, current physical activity during leisure time, dietary intakes, education, and medical history. Height and weight were measured according to standard protocols once at baseline attendance for each participant and body mass index (BMI) was calculated.

### Statistical analysis

The characteristics of study participants were compared among those with THR for OA, those with THR for #NOF, and those with no THR, using one way analysis of variance (ANOVA) for continuous variables and chi-squared test for categorical variables. Binary logistic regression models were used to estimate the odds ratio (OR) and 95% confidence intervals (CI) for any THR due to hip OA or #NOF in relation to AMD (early, intermediate and late AMD), using those with no AMD as the reference group. Age, sex, BMI, country of birth, and smoking status were included in all models. Visual acuity was not recorded hence we were unable to include this in our analyses. Other potential confounding variables were included in the analyses if they changed the OR by more than 5%. These included education level, multivitamin supplement, physical activity, alcohol consumption, and history of stroke, hypertension, cardiovascular disease or diabetes. None of these variables changed the OR by more than 5%, and were not included in the final regression models. All statistical analyses were performed using Stata 12.0 (StataCorp LP, College Station, TX, USA).

## Results

The characteristics of the 20,744 participants are presented in Table 1. There were 4,267 persons with early AMD, 2,678 with intermediate AMD and 118 with late AMD. AMD prevalence of 20.6% (95% CI 20.0%, 21.1%) for early, 12.9% (95% CI 12.5%, 13.4%) for intermediate AMD and 0.6% (95% CI 0.5%, 0.7%) for late AMD was comparable with the respective categories in the Melbourne population-based study on AMD prevalence[25]. 791 (3.8%) THR due to OA and 102 (0.5%) THR due to #NOF were identified. Persons who had THR for #NOF (mean age 73.3 years) tended to be older than those who had THR for OA (mean age 68.6 years) (p<0.001). There were more females in the group with THR for #NOF (p<0.001) and more persons born in Australia or UK in the group with THR for OA (p<0.001). Smoking status (p = 0.12) and BMI (p = 0.46) did not differ between groups.

**Table 1:** Characteristics of study participants.

	THR due to OA (n = 791)	THR due to #NOF (n = 102)	No THR (n = 19,851)	P value
Age at photo (years)	68.6 (7.8)	73.3 (6.5)	64.9 (8.7)	<0.001
Female, n (%)	512 (64.7)	77 (75.5)	11,893 (59.9)	<0.001
Body mass index (kg/m^2^)	26.9 (4.2)	26.0 (3.8)	26.1 (4.1)	0.46
Country of birth, n (%)				<0.001
Australia/UK	746 (94.3)	90 (88.2)	17,213 (86.7)	
Italy/Greece	45 (5.7)	12 (11.8)	2,638 (13.3)	
Smoking, n (%)				0.12
Non-smoker	467 (59.0)	62 (60.8)	12,032 (60.6)	
Former smoker	271 (34.3)	29 (28.4)	6,109 (30.8)	
Current smoker	53 (6.7)	11 (10.8)	1,708 (8.6)	
Age-related macular degeneration (AMD)				<0.001
No AMD	508 (64.2)	57 (55.9)	13,116 (66.1)	
Early AMD	148 (18.7)	20 (19.6)	4,099 (20.7)	
Intermediate AMD	132 (16.7)	18 (17.7)	2,528 (12.7)	
Late AMD	3 (0.4)	7 (6.9)	108 (0.5)	

Data presented as mean (SD) or number (%)

Table 2 shows AMD and its association with THR. After adjustment for age, sex, BMI, smoking, and country of birth, persons with intermediate AMD had an increased cumulative 10-year incidence of THR for OA compared with those without AMD (OR 1.22, 95% CI 1.00–1.49), but there were no associations with the early stage of AMD or late AMD. Persons with late AMD were at higher risk of THR due to #NOF compared with those without AMD (OR 5.21, 95% CI 2.25–12.02). No significant association was observed for early or intermediate AMD and #NOF. There was no evidence for an effect modification by sex on the association between prevalent AMD and cumulative 10-year incidence of THR. The results were similar when additional adjustment was performed for medical history (diabetes, hypertension and atherosclerosis), lifestyle factors (physical activity and alcohol consumption), use of vitamin supplements and nonsteroidal anti-inflammatory drugs, and education (data not shown).

**Table 2:** Relationship of AMD with risk of total hip replacement for osteoarthritis and fractured neck of femur.

	Univariable analysis		Multivariable analysis*	
	Odds ratio	P value	Odds ratio	P value
	(95% CI)		(95% CI)	
**Total hip replacement for osteoarthritis**				
No AMD	1.00		1.00	
Early AMD	0.93 (0.77, 1.12)	0.46	1.02 (0.85, 1.24)	0.81
Intermediate AMD	1.35 (1.11, 1.64)	0.003	**1.22 (1.004, 1.49)**	**0.046**
Late AMD	0.72 (0.23, 2.27)	0.57	0.44 (0.14, 1.39)	0.16
**Total hip replacement for fractured neck of femur**				
No AMD	1.00		1.00	
Early AMD	1.12 (0.67, 1.87)	0.66	1.26 (0.75, 2.11)	0.38
Intermediate AMD	1.64 (0.96, 2.79)	0.07	1.17 (0.69, 2.01)	0.56
Late AMD	14.91 (6.65, 33.44)	<0.001	**5.21 (2.25, 12.02)**	**<0.001**

*adjusted for age, gender, body mass index, smoking, and country of birth

## Discussion

We found a direct association between AMD and the 10-year cumulative incidence of THR due to OA and #NOF in the elderly. Intermediate AMD was directly associated with an increased 10-year incidence of THR due to OA, and late AMD was directly associated with increased 10-year incidence of THR for #NOF.

The association between AMD and the risk of hip OA has not been examined previously. We found an increased 10-year cumulative incidence of THR due to OA with intermediate AMD. This relationship was independent of age, sex, BMI, lifestyle factors (physical activity, smoking, alcohol consumption, and vitamin supplements) and medical history (diabetes, hypertension, atherosclerosis, and use of nonsteroidal anti-inflammatory drugs). Chronic inflammation is thought to play an important role in the pathogenesis of both AMD and OA[11–13,16,17], and these conditions may potentially share common inflammatory pathways. Inflammation-related genes have been associated with OA[15]. In OA, there is marked hyperplasia of the synovial lining cells with an infiltration of inflammatory cells mainly of macrophages but also T and B cells[26], mast cells[27] and natural killer cells[28]. Inflammatory mediators have also been found in OA synovial fluid as well as serum[14,29]. It has been shown that inflammation is present in OA joints well before the development of significant radiographic changes[17], with overexpression of inflammatory mediators in early rather than late disease[30]. The hip biomechanics (hip bone shape and pressure points) may also contribute to an increased risk of OA.

In the early and intermediate stages of AMD, drusen which are deposits located below the retinal pigment epithelium (RPE), have been postulated to be a by-product of a localised inflammatory response following RPE and Bruch’s Membrane injury involving the complement system, or themselves drive inflammation due to the abnormal lipid rich deposits[31,32]. Inflammatory cells and infiltrates, including components of the activated complement cascade, have been found in both drusen and in Bruch’s Membrane[31,32]. Genetic studies have found a strong association of polymorphisms in the complement factor H (*CFH*) gene (Y402H) with AMD; where *CFH* is an inhibitor of the alternative complement pathway[10]. Hence the activation of the immune system is a central feature of both OA and AMD[11,17]. After controlling for potential confounders, the relationship between intermediate AMD and the risk of OA-related THR was statistically significant although there were no significant associations with early or late AMD. There were too few cases of late AMD (n = 3 for THR for OA) to find an association between late AMD and risk of OA-related THR. It could be postulated that the systemic inflammatory disease processes may have been too early in early AMD cases to observe chronic associations. Our data showed an association which needs further exploration to evaluate potential common pathways as this may shed further light into their pathogenesis.

We did however, demonstrate that late AMD was associated with a 5.2 fold increased 10-year cumulative incidence of primary THR for #NOF. A US study using a 5% random sample of Medicare beneficiaries from 1996–1999, which included 8,596 coded cases of exudative AMD, 26,942 coded cases of atrophic AMD, and 1,013,748 cases without AMD codes, reported a 4.6% 4-year cumulative incidence (1995–1999) of hip fracture. This study found atrophic AMD to be directly associated with an 11% increase in the four-year odds of hip fracture (OR 1.11, 95% CI 1.06 to 1.16, P >0.001), but there were no associations with exudative AMD (OR 1.03, 95% CI 0.95, 1.12)[18]. The accuracy of the International Classification of Diseases taxonomy coding in the Medicare database has been reported to be high for hip fractures and exudative AMD, however the accuracy of atrophic AMD was undetermined. Of note, in the United States, the Medicare health insurance programme does not have the same coverage as in Australia. The authors postulated that miscoding error could have biased the results towards null. A similar American longitudinal retrospective cohort study using 5% sample Medicare claims data found elderly individuals with newly diagnosed AMD (91.5% of AMD was coded dry or unspecified) in 1994 had higher rates of hip fracture than those without AMD during a 10-year follow-up period, OR 1.09 (1.04–1.14)[19].

It is well documented that reduced visual acuity is associated with a 30–90% increased odds of hip fracture[33]. It is estimated that over 95% of #NOF in the elderly are the result of a fall[1]. It is likely that AMD, particularly in its late stages, increases the risk of falls through impaired vision. Our findings of an association with vision-threatening late AMD and high risk of #NOF is likely to relate to falls due to poor visual acuity (anti-VEGF treatment was not available at the time when AMD was determined in our study), although we are not able to directly test this as acuity information was not collected. It is not surprising that an association between early or intermediate AMD was not found, as visual acuity is not typically affected.

Strengths of our study include the large sample size in one comprehensive study that provided AMD, OA and #NOF data. AMD status was accurately determined via colored fundus photography, and THR cases ascertained directly from the AOA NJRR. The AOA NJRR data is validated and nearly complete in terms of joint replacement performed in Australia. Detailed AMD grading was performed on high-resolution digital color fundus images, which was the most appropriate methodology during the period before intra-vitreal anti-VEGF treatment for neovascular AMD was commonly available. Our study also has some limitations. The fact that AMD was assessed within the THR follow-up time frame limited the assessment of a temporal relationship between AMD and THR risk. AMD status was obtained at only one time point (2003–2007) and data on THR were collected between 2001 and 2011 during which time the AMD status could have changed. The number of participants with late AMD was small, and we had no data on visual acuity, or bone mineral density to include in our analyses. For #NOF, the registry only identifies the patients who have #NOF treated by THR which is estimated to be about 1/3 of all #NOF in Australia[5].

Our findings have both clinical and public health implications. The association of intermediate AMD with the risk of THR due to OA suggests that similar underlying mechanisms and therefore genetic risk profile may exist for both chronic inflammatory conditions, and this association warrants further research. If common inflammatory pathways were present, this can be targeted as a treatment to reduce inflammatory mediators for both diseases. Additionally, we recognize that late AMD is strongly associated with the risk of #NOF, with fractures often associated with falls. More work is needed to optimize vision to reduce this risk of falls and fractures in this high risk group as along with issues surrounding the treatment of #NOF. It is well recognized that mortality rates after # NOFs are much higher than that in the general population[34]. According to the Australian Macular Degeneration Foundation, there are an estimated 20,734 new cases neovascular AMD annually (based on incidence figures from the Blue Mountains Eye Study[35]). Neovascular AMD is now treatable by regular intravitreal injections of anti-VEGF agents. It is important that vision loss from neovascular AMD is minimized by early assessment and timely treatment before scarring occurs and vision loss becomes irreversible. Early detection of neovascular AMD and rapid treatment with anti-VEGF can potentially reduce the risk of hip fracture in the elderly and the associated health care burden.

## Conclusions

The association between intermediate AMD and an increased 10-year incidence of THR due to OA suggests the possibility of similar inflammatory processes underlying both chronic diseases. The association of late AMD with an increased 10-year incidence of THR due to #NOF may be due to an increased prevalence of fractures in those with poor central vision associated with the late complications of AMD.
